# Leiomyosarcoma of the Renal Vein That Enlarged Laterally While Preserving the Structure of the Renal Vein Lumen

**DOI:** 10.1002/iju5.70273

**Published:** 2026-10-07

**Authors:** Naoki Tashiro, Yume Sakaki, Akito Fujinaga, Takahiro Nagai, Chie Onizuka, Shoichiro Mukai, Yoshiko Umekita, Yuichiro Sato, Toshiyuki Kamoto, Atsuro Sawada

**Affiliations:** ^1^ Department of Urology, Faculty of Medicine University of Miyazaki Miyazaki Japan; ^2^ Section of Oncopathology and Morphological Pathology, Department of Pathology, Faculty of Medicine University of Miyazaki Miyazaki Japan

**Keywords:** leiomyosarcoma, preserve vein lumen, renal vein

## Abstract

**Introduction:**

Primary renal vein leiomyosarcoma is an extremely rare disease and the tumor often infiltrates the vessel wall. We report a case of primary renal vein leiomyosarcoma that enlarged while preserving the structure of the renal vein lumen.

**Case Presentation:**

A 64‐year‐old woman underwent a computed tomography scan, which revealed a left renal hilar mass. Extra‐gastrointestinal stromal tumor, leiomyosarcoma, and inflammatory myofibroblastic tumor remained in the differential diagnoses based on blood, urine, and imaging test findings. The tumor, left renal vein, kidney, and adrenal gland were resected. Hematoxylin–eosin staining revealed a tumor arising from the renal vein wall and proliferating outward beyond the vessel. The diagnosis was primary renal vein leiomyosarcoma.

**Conclusion:**

When a well‐defined solid tumor adjacent to the renal vein is identified, leiomyosarcoma should be considered in the differential diagnosis, and complete resection of the tumor should be pursued.

## Keynote Message

Complete resection is required for leiomyosarcoma of the renal vein, and the presence of inferior vena caval luminal extension has been reported as a risk factor for recurrence. Given the possibility of early resection at a stage where there is no invasion into the venous lumen, as seen in this case, aggressive resection should be performed when this disease is suspected.

## 1 Introduction

Primary renal vein leiomyosarcoma is an extremely rare disease, and its diagnosis is often challenging due to a lack of specific imaging findings. We report a case of a smooth muscle tumor that enlarged laterally while preserving the structure of the renal vein lumen.

## 2 Case Report

A 64‐year‐old woman underwent a contrast‐enhanced computed tomography (CECT) scan at her previous gynecologist's practice as part of cervical cancer screening. At that time, a left renal hilar mass was incidentally noted. Following magnetic resonance imaging (MRI) and positron emission tomography (PET)‐CT, the patient was referred to our department for further investigation and treatment. A cervical conization procedure for cervical cancer was performed prior to referral to our department. Blood tests were unremarkable, and multiple tumor markers were negative. A urine test was negative for all three catecholamine fractions (Table 1). CECT demonstrated an irregular mass within the left kidney, exhibiting internal heterogeneity with mild peripheral enhancement (Figure 1). MRI revealed a tumor with low T1‐weighted signal intensity, internal heterogeneity on T2‐weighted imaging, and diffusion restriction (Figure 2).

**TABLE 1:** Blood test and urinary biochemical analysis.

Blood biochemistry			Serology				Urinary biochemical analysis		
						**Range**			
TP	7.30	g/dL	CEA	1.9	ng/mL	(0.0–5.0)	Ad	0.02	ng/mL
Alb	4.5	g/dL	AFP	5	mg/mL	(0.0–7.0)	Nad	0.17	ng/mL
Glu	103	mg/dL	CA19‐9	29.5	U/mL	(0.0–37.0)	DOA	≦ 0.02	ng/mL
BUN	10.4	mg/dL	CA125	11.4	U/mL	(0.0–35.0)			
Cre	0.64	mg/dL	SCC	0.7	mg/mL	(0.0–2.3)			
Na	145	mEq/L	sIL‐2R	452	U/mL	(157–474)			
Cl	105	mEq/L	HBs Ag	—					
K	4.0	mEq/L	HCV Ab	—					
AST	17	U/L	TP Ab	—					
ALT	8	U/L	**Coagulation**						
LD	179	U/L	PT	11.6	s				
ALP	47	U/L	PT‐INR	0.92					
γ‐GTP	14	U/L	APTT	25.6	s				
CRP	0.02	mg/dL							

*Note:* Blood tests were unremarkable, and multiple tumor markers were negative. A urine test was negative for all three catecholamine fractions.

**FIGURE 1:**
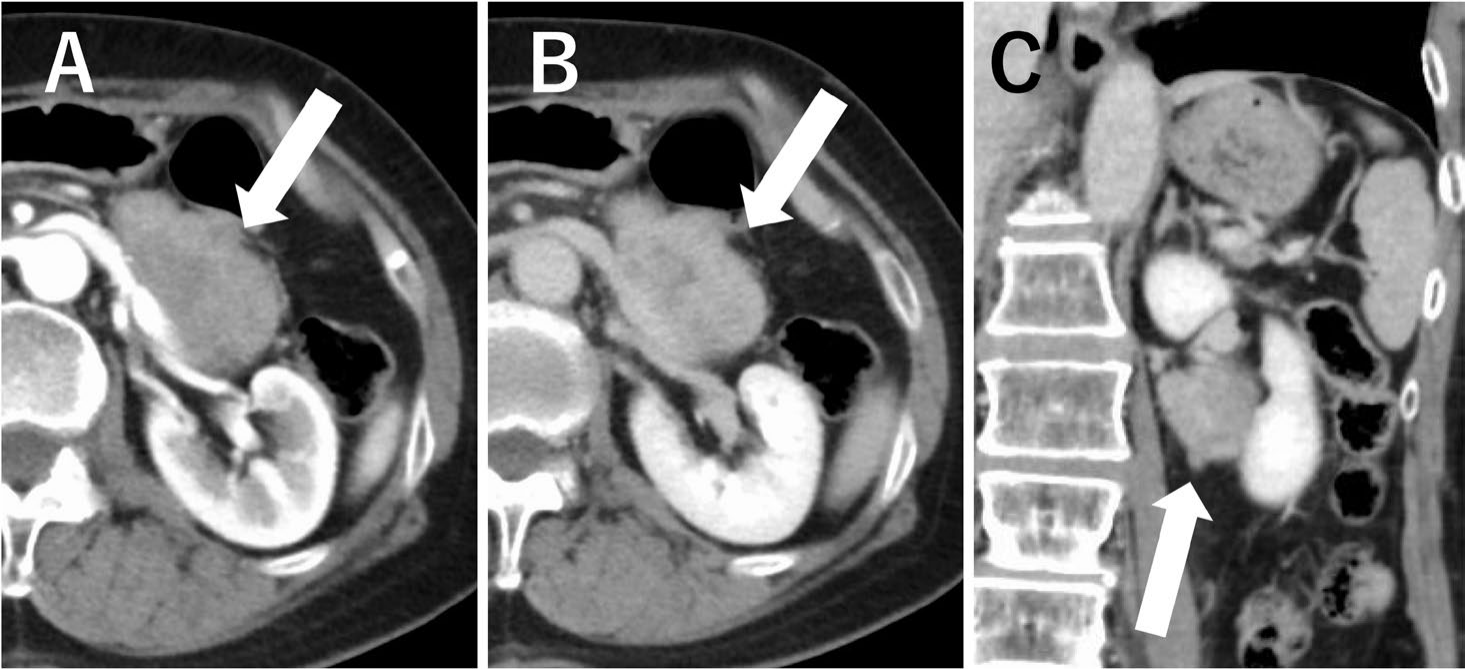
Contrast‐enhanced CT. (A) Axial Arterial Phase. (B) Axial Delayed Phase. (C) Coronal image. A well‐defined, irregularly shaped mass with mildly enhanced margins and internal heterogeneous enhancement was noted on the medial aspect of the left kidney, extensively contacting the renal vein. It became more intensely stained during the excretion phase.

**FIGURE 2:**
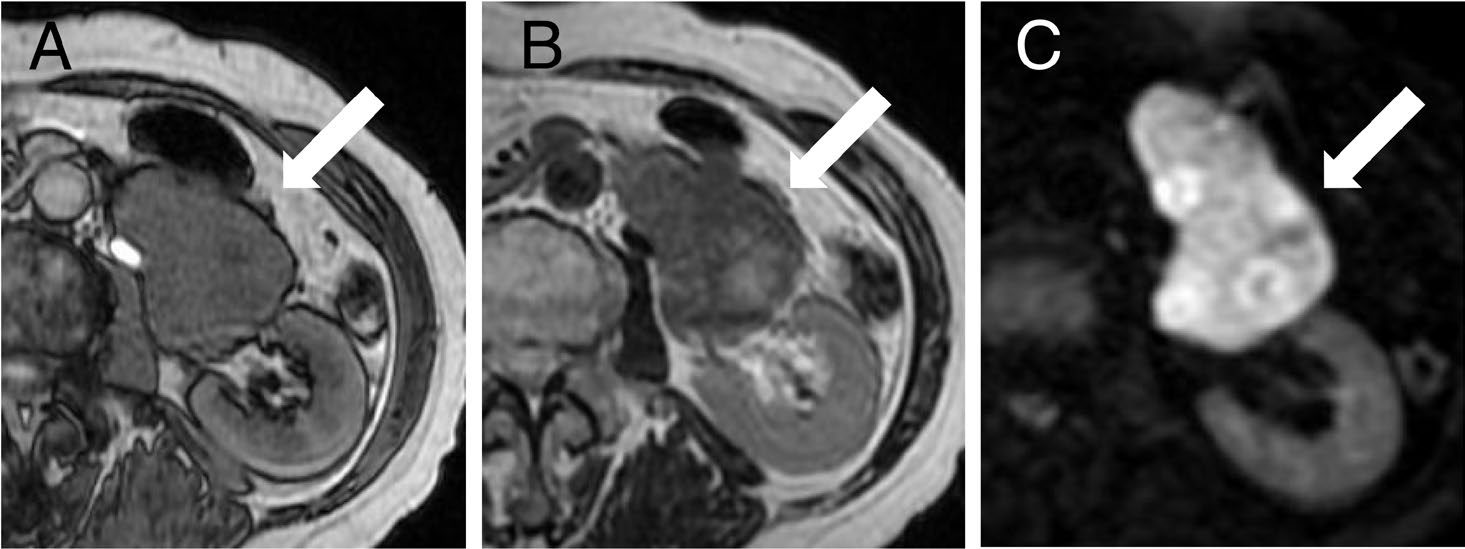
MRI. (A) T1 weighted image. (B) T2 weighted image. (C) Diffusion‐weighted imaging. It exhibited low signal intensity on T1‐weighted images, and on T2‐weighted images, low and high signal intensities were present in an uneven mixture within the lesion. Diffusion‐weighted imaging revealed diffusion restriction. There was no clear continuity between the kidneys, adrenal glands, pancreas, and the tumor.

Given its location at the renal hilum, the absence of clear continuity with the gastrointestinal tract, poor contrast enhancement, MRI signal characteristics, the absence of clear flow void, weak FDG‐PET uptake, and blood test, extra‐gastrointestinal stromal tumor, leiomyosarcoma, and inflammatory myofibroblastic tumor remained in the differential diagnoses. Due to the tumor's extensive contact with the left renal vein, preservation of the left kidney was deemed impractical. A laparoscopic left renal hilar tumor resection and nephrectomy were performed. Surgery was performed under general anesthesia following standard procedures for laparoscopic left nephrectomy. The tumor near the renal hilum protruded inward significantly, and it was extensively and firmly adherent to the renal vein. The left renal vein was transected more centrally than the tumor, and the tumor and left kidney were resected en bloc. Due to the proximity of the left adrenal gland to the tumor, the adrenal gland was also resected. The tumor measured 60 mm × 53 mm. Its cut surface was white and lobulated. The tumor protruded laterally from the renal vein. Hematoxylin–eosin staining revealed a tumor arising from the renal vein wall and proliferating outward beyond the vessel. The tumor cells showed spindle‐shaped nuclei with eosinophilic cytoplasm, elongated tobacco‐seed‐like nuclei, high cellularity, and focal necrosis. Immunohistochemically, the tumor cells are positive for desmin, h‐caldesmon, and SMA, whereas negative for S‐100 and CD34 (Figure 3). Ki‐67 labeling index is 17%. FNCLCC Grade is 1 (total score 2). Based on the above findings, the diagnosis was primary renal vein leiomyosarcoma. The tumor did not invade the renal parenchyma, and the resection margins were clear. This patient has remained free of recurrence for 29 months after surgery.

**FIGURE 3:**
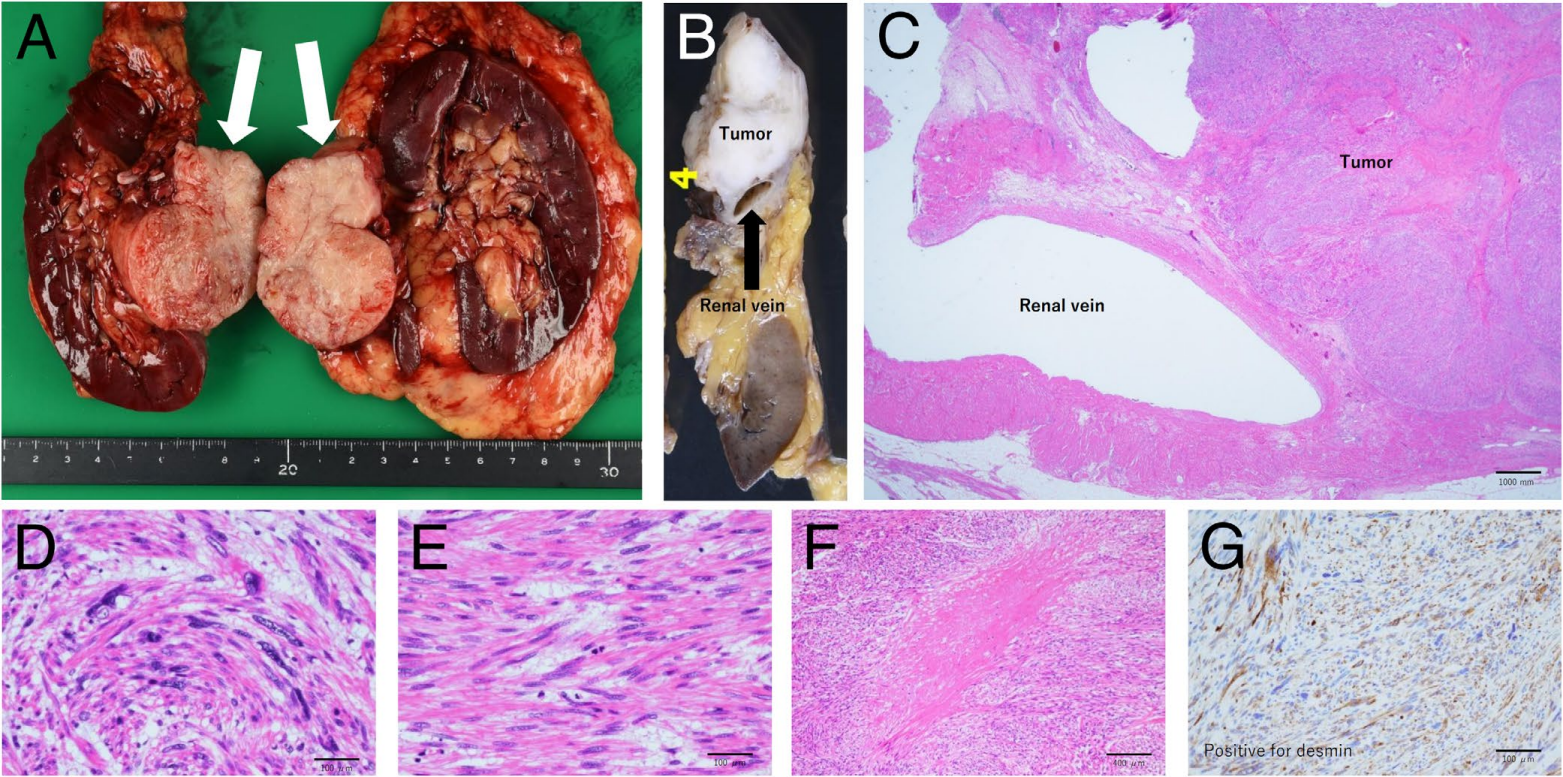
Macroscopical finding and pathological histological findings. (A) 60 mm × 53 mm, its cut surface was white and lobulated. (B) The tumor protruded laterally from the renal vein. (C) Proliferating outward from the renal vein wall (×40). (D) The tumor cells showed spindle‐shaped nuclei with eosinophilic cytoplasm. (E) Few mitotic figures are present (2/10 HPF). (F) Necrosis was observed internally. (G) Positive for desmin staining.

## 3 Discussion

Leiomyosarcoma of the renal vein is most common in patients in their 60s [1]. CT findings include internal necrosis and mild to moderate contrast enhancement, but no characteristic findings were observed [2]. MRI findings typically include high signal on T1‐weighted images compared to the kidneys, and slightly elevated signal on T2‐weighted images that is considered to be lower than that of the kidneys [3].

This case involved a female patient in her 60s, the age group most commonly affected. MRI findings are all compatible with primary renal vein leiomyosarcoma. However, atypical features, such as the absence of necrosis or cystic changes on CT, made it difficult to diagnose this patient preoperatively. It is important to keep this disease in mind as one of the differential diagnoses when certain characteristics are present.

The primary treatment for leiomyosarcoma is surgical resection. If the tumor has infiltrated adjacent organs, active resection of the affected organs is recommended. Active surgical intervention is also recommended for recurrence of the tumor [4].

In patients with vascular smooth muscle sarcoma, reports indicate that the tumor infiltrates the vessel wall, leading to the formation of tumor emboli and obstruction of the lumen. Searching PubMed, Medical Online, and the Japanese Medical Journal using the keywords “Leiomyosarcoma” and “renal vein” identified a total of 77 cases of primary renal vein leiomyosarcoma. Of these, 67 were summarized by Novak et al. [1] and an additional 10 cases were subsequently reported [2, 5, 6, 7, 8, 9, 10, 11, 12]. Among the reports accessible under the current search criteria, there were two patients where the renal vein lumen was considered preserved [6, 13], while 14 patients experienced clear intravascular invasion [2, 3, 5, 8, 9, 12, 14, 15, 16, 17, 18, 19]. The proportion with invasion of the renal vein was high.

Novak et al. [1] identified the presence of inferior vena caval (IVC) luminal extension and tumor malignancy as factors contributing to local recurrence rates. Because FNCLCC grade is 1 and there was no IVC luminal extension in this case, the risk of local recurrence is low.

The tumor in this patient presented with a relatively uncommon growth pattern wherein the tumor protruded outward while maintaining its vascular lumen without invading the renal vein lumen. We also speculate this growth pattern may have contributed to the fact that the patient remains recurrence‐free to this day.

## 4 Conclusion

We report a case of leiomyosarcoma that originated in the renal vein, which is rare among leiomyosarcomas. Although preoperative diagnosis is challenging, when a well‐defined solid tumor adjacent to the renal vein is identified, renal vein primary leiomyosarcoma should be considered in the differential diagnosis, and complete resection of the tumor should be pursued.

## Funding

The authors have nothing to report.

## Ethics Statement

The authors have nothing to report.

## Consent

The requirement for obtaining informed consent was waived due to the retrospective observational study and was obtained in an opt‐out format.

## Conflicts of Interest

The authors declare no conflicts of interest.

## Data Availability

Data sharing not applicable to this article as no datasets were generated or analysed during the current study.
